# Great phenotypic and genetic variation among successive chronic *Pseudomonas aeruginosa* from a cystic fibrosis patient

**DOI:** 10.1371/journal.pone.0204167

**Published:** 2018-09-13

**Authors:** Carmen Lozano, José Manuel Azcona-Gutiérrez, Françoise Van Bambeke, Yolanda Sáenz

**Affiliations:** 1 Area de Microbiología Molecular, Centro de Investigación Biomédica de La Rioja (CIBIR), Logroño, Spain; 2 Departamento de Diagnóstico Biomédico, Laboratorio de Microbiología, Hospital San Pedro, Logroño, Spain; 3 Pharmacologie Cellulaire et Moléculaire, Louvain Drug Research Institute, Université Catholique de Louvain, Brussels, Belgium; Universidad Nacional de la Plata, ARGENTINA

## Abstract

**Background/Objectives:**

Different adapted *Pseudomonas aeruginosa* morphotypes are found during chronic infections. Relevant biological determinants in *P*. *aeruginosa* successively isolated from a cystic fibrosis (CF) patient were analyzed in this work to gain insight into *P*. *aeruginosa* heterogeneity during chronic infection.

**Methods:**

Seventeen *P*. *aeruginosa* isolates collected from a patient over a 3 year period were included, 5 small colony variants (SCV) and 12 mucoids. The following analyses were performed: Pulsed-Field-Gel-Electrophoresis (PFGE)/Multilocus- sequence-typing (MLST)/serotype, antimicrobial susceptibility, growth curves, capacity to form biofilm, pigment production, elastase activity, motility; presence/expression of virulence/quorum sensing genes, and identification of resistance mechanisms.

**Results:**

All isolates had closely related PFGE patterns and belonged to ST412. Important phenotypic and genotypic differences were found. SCVs were more resistant to antimicrobials than mucoid isolates. AmpC hyperproduction and efflux pump activity were detected. Seven isolates contained two integrons and nine isolates only one integron. All SCVs showed the same OprD profile, while three different profiles were identified among mucoids. No amino acid changes were found in MutL and MutS. All isolates were slow-growing, generally produced high biofilm, had reduced their toxin expression and their quorum sensing, and showed low motility. Nevertheless, statistically significant differences were found among SCV and mucoid isolates. SCVs grew faster, presented higher biofilm formation and *flicA* expression; but produced less pyorubin and pyocyanin, showed lower elastase activity and *rhlR*, *algD*, and *lasB* expression than mucoid isolates.

**Conclusion:**

These results help to understand the molecular behavior of chronic *P*. *aeruginosa* isolates in CF patients.

## Introduction

*Pseudomonas aeruginosa* is one of the most important causes of nosocomial infection. Its prevalence is very high in patients with cystic fibrosis (CF), in which innate immunity is compromised and the mucus properties are favorable to bacterial colonization. The respiratory tract of these patients is colonized during childhood or adolescence, and remains chronically infected, generally by a single *P*. *aeruginosa* lineage [1,2]. Important phenotypic and genetic differences have been found in isolates obtained at an early stage and those detected during chronic infections [3–6]. During early phases of infection, *P*. *aeruginosa* shows high virulence factors expression and is generally susceptible to antibiotics, whereas at chronic stage, it reduces the expression of toxins and its motility, is more resistant to antimicrobial agents, exhibits mucoid phenotype, presents reduced quorum sensing, increased mutation rates, and/or has enhanced biofilm formation capacity [3–6]. Diverse adapted morphotypes have been found through late stage [7]. Two main colony morphologies, mucoid and small colony variant (SCV), have been described, although other types (smooth, rough, colorless…) have also been identified [6–8]. It is thought that these diverse populations evolve along the time in order to be adapted to selective pressure in CF airways (co-infecting species, deficient oxygen and nutrient availability, inflammatory responses, oxidative stress or presence of antibiotics), generating different subpopulations that coexist within the patient [9].

All these biological factors make it very difficult to eradicate *P*. *aeruginosa* at late stages. A deeper understanding of the genotypic differences of these chronic adapted isolates, of how they behave phenotypically and of their antimicrobial resistance mechanisms would be very useful to provide tools for a better approach of treating infections in CF patients.

The aim of this study was to analyze relevant biological determinants in *P*. *aeruginosa* isolates collected successively from a CF patient over a 3 year period in order to gain insight into their heterogeneity during chronic infection.

## Materials and methods

### Bacterial isolates

*P*. *aeruginosa* isolates from the same 46-year-old CF patient were collected as a part of routine testing in San Pedro Hospital of La Rioja (Northern Spain). The first *P*. *aeruginosa* isolate from this patient dated from 2006; however our hospital started collecting them since 2012. Written informed consent was obtained from the patient to carry out this analysis. A total of 17 *P*. *aeruginosa* isolates obtained from April-2012 to June-2015 were selected. Two distinct colony morphologies were identified: five isolates were SCV, while the remaining 12 isolates showed mucoid phenotype (Table 1).

**Table 1:** Information about the 17 *P*. *aeruginosa* isolates obtained from the same patient.

Isolate	Date sample	Sample	Control/Exacerbation	Colony morphology	PFGE pattern
Ps599	23/04/2012	Sputum	Exacerbation	Mucoid	P1c
Ps600	11/10/2012	Sputum	Control	Mucoid	P1b
Ps601	19/10/2012	Sputum	Exacerbation	Mucoid	P1c
Ps602	19/10/2012	Sputum	Exacerbation	SCV	P1e
Ps270	21/06/2013	Sputum	Exacerbation	SCV	P1h
Ps338	18/09/2013	Sputum	Exacerbation	Mucoid	P1d
Ps339	18/09/2013	Sputum	Exacerbation	SCV	P1g
Ps603	26/12/2013	Sputum	Exacerbation	Mucoid	P1e
Ps604	04/09/2014	Sputum	Exacerbation	Mucoid	P1c
Ps605	18/12/2014	Sputum	Control	Mucoid	P1c
Ps606	25/02/2015	Sputum	Exacerbation	Mucoid	P1a
Ps607	09/03/2015	Sputum	Exacerbation	Mucoid	P1c
Ps608	23/03/2015	Tracheal aspirate	Exacerbation	SCV	P1f
Ps684	07/04/2015	Bronchial aspirate	Exacerbation	Mucoid	P1c
Ps685	14/04/2015	Sputum	Exacerbation	Mucoid	P1a
Ps686	25/06/2015	Sputum	Control	SCV	P1g
Ps683	02/07/2015	Sputum	Control	Mucoid	P1b

SCV: small colony variant

### Molecular typing

Pulsed-Field-Gel-Electrophoresis (PFGE) and Multilocus-sequence-typing (MLST) (http://pubmlst.org/paeruginosa/) were carried out on all isolates [10]. PFGE patterns were analyzed by the Java program GelJ using the Dice coefficient [11], and according to Tenover criteria [12]. Serotype identification was performed with monovalent antisera specific for 16 different *P*. *aeruginosa* O serotypes (Bio-Rad, Marnes-la-Coquette, France).

### Antimicrobial susceptibility

Susceptibility testing to ticarcillin, piperacillin, piperacillin/tazobactam, ceftazidime, cefepime, aztreonam, imipenem, meropenem, doripenem, gentamicin, tobramycin, amikacin, netilmicin, ciprofloxacin, temocillin, and colistin was carried out by disc-diffusion agar method [13]. Double-disc synergy methods were used for detecting ESBL, MBL, and class A carbapenemase phenotypes [14]. Minimum inhibitory concentration (MIC) of imipenem was determined by E-test. AmpC hyperproduction was determined by phenotypic test using ceftazidime discs and Mueller Hinton (MH) agar plates in the presence or absence of 250 mg/L of cloxacillin (no toxicity detected at this concentration). Efflux pump activity was investigated using ticarcillin, imipenem, meropenem, gentamicin, ciprofloxacin, and norfloxacin in presence/absence of 20 mg/L of the broad-spectrum inhibitor Phe-Arg-β-naphthylamide (PAβN) (the highest non-toxic concentration tested without effect on bacterial growth). The results were analyzed as previously recommended [15].

### Characterization of porin OprD and RND efflux pumps

Mutations in *oprD* and efflux pump genes, *oprD* promoter and efflux regulatory genes were examined by PCR and sequencing (see S1 Table) [10,16]. The membrane protein profiles of selected isolates were analyzed by SDS-PAGE as previously described [17]. Additionally, the *oprD* gene expression was studied by RT-qPCR (as explained below).

### Determination of integron structures

Genes encoding type 1 and 2 integrases and the 3’ conserved segment was studied by PCR. The class 1 promoters (Pc) and variable regions were analyzed by PCR and sequencing [10,18,19].

### Presence of virulence genes and DNA mismatch repair system genes

The presence of *exoS*, *exoU*, *exoY*, *exoT*, *exoA*, *lasA*, *lasB*, *aprA*, *rhlAB*, *rhlI*, *rhlR*, *lasI*, and *lasR* genes was studied by PCR [15]. The *mutL* and *mutS* genes were analyzed by PCR and sequencing [5].

### Growth curves and generation time determination

Fifty μL of an overnight LB broth culture was transferred to fresh LB broth (50 mL) and incubated at 37°C with shaking at 120 rpm for 24 h. OD_620nm_ for each isolate were measured over time. Colony Forming Units (cfu) per mL were determined by seeding duplicated serial dilutions (10^−4^, 10^−6^, and 10^−8^) of each sample onto Brain Heart Infusion (BHI) agar plates. Generation time (GT) was calculated during logarithmic phase.

### Biofilm quantification

Biofilm assays were performed by crystal violet (CV) staining to analyze total biofilm biomass, and by fluorescein diacetate (FDA) assay to study the bacterial metabolic activity inside the biofilm structure. Both methods were performed in microtiter 96-well plates using an initial 10^6^ cfu/mL inoculum, and measured after 24h of incubation as previously recommended [20]. Measures were performed using a POLARstar Omega microplate reader (BMG Labtech).

### Elastase and pigment production

Elastase activity was determined by the Elastin-Congo-Red assay [21]. The chloroform-extract method was used for pyocyanin and pyorubin pigments quantification [22].

### Motility

Swarming and swimming motility were studied as previously described with modifications [23]. Isolates were grown in 5 mL of LB broth at 37°C with shaking until OD_620nm_ of 0.8 (1x10^9^ cells), and 4 μL were placed on the middle of 0.5% (swarming) and 0.3% (swimming) LB agar plates. After incubation at 37°C during 20 h, the plates were imaged with Chemi Doc system (Bio-Rad), and processed with Image Lab software (version 5.2.1, Bio-Rad).

### RNA extraction and RT-qPCR

The expression of *ampC*, *oprD*, *algD*, *rhlR*, *lasR*, *lasB*, *pslA*, *pelA*, *exoS*, *exoT*, *pcrV*, *popB*, *popD*, and *flicA* genes was studied by RT-qPCR. Isolates were grown in LB broth until an OD_620nm_ of 0.2–0.4, and 1 mL culture was added to a tube containing 125 μL of stop solution (95% ethanol/ 5% phenol). Each tube was treated with lysozyme (100 μg) at room temperature for 20 min. Total RNA was extracted using RNeasy Mini Kit (QIAGEN) and treated with DNase (DNA-free, Ambion). The absence of contaminating DNA was checked by conventional PCRs. The RNA concentration was measured using a NanoDrop ND-1000 V3.7.1. cDNA was synthesized using Precision nanoScript Reverse Transcription kit (PrimerDesign) and qPCR was performed with an ABI 7300 Real-Time PCR System (Applied Biosystems) using Power SYBR green PCR master mix (Applied Biosystems). The primers used are shown in S1 Table [24–29]. Relative gene expression was calculated by 2^-ΔΔCT^ method. The *rpsL* gene was used as reference housekeeping gene.

### Control isolates

*P*. *aeruginosa* PAO1 strain was included as control in all assays. In the case of the *flicA* expression analysis (not expressed by PAO1), *P*. *aeruginosa* CHA strain was used as calibrator. All assays were performed at least in triplicate.

All sequences obtained during this work were compared with the genome of *P*. *aeruginosa* PAO1 strain (GenBank accession no. AE004091).

### Statistical analysis

GraphPad Prism (version 6.01) from GraphPad Software (San Diego, California) was used for graphical representations, and R-commander program (version 2.2–1) for statistical analyses.

## Results

### Molecular typing

**Fig 1:**
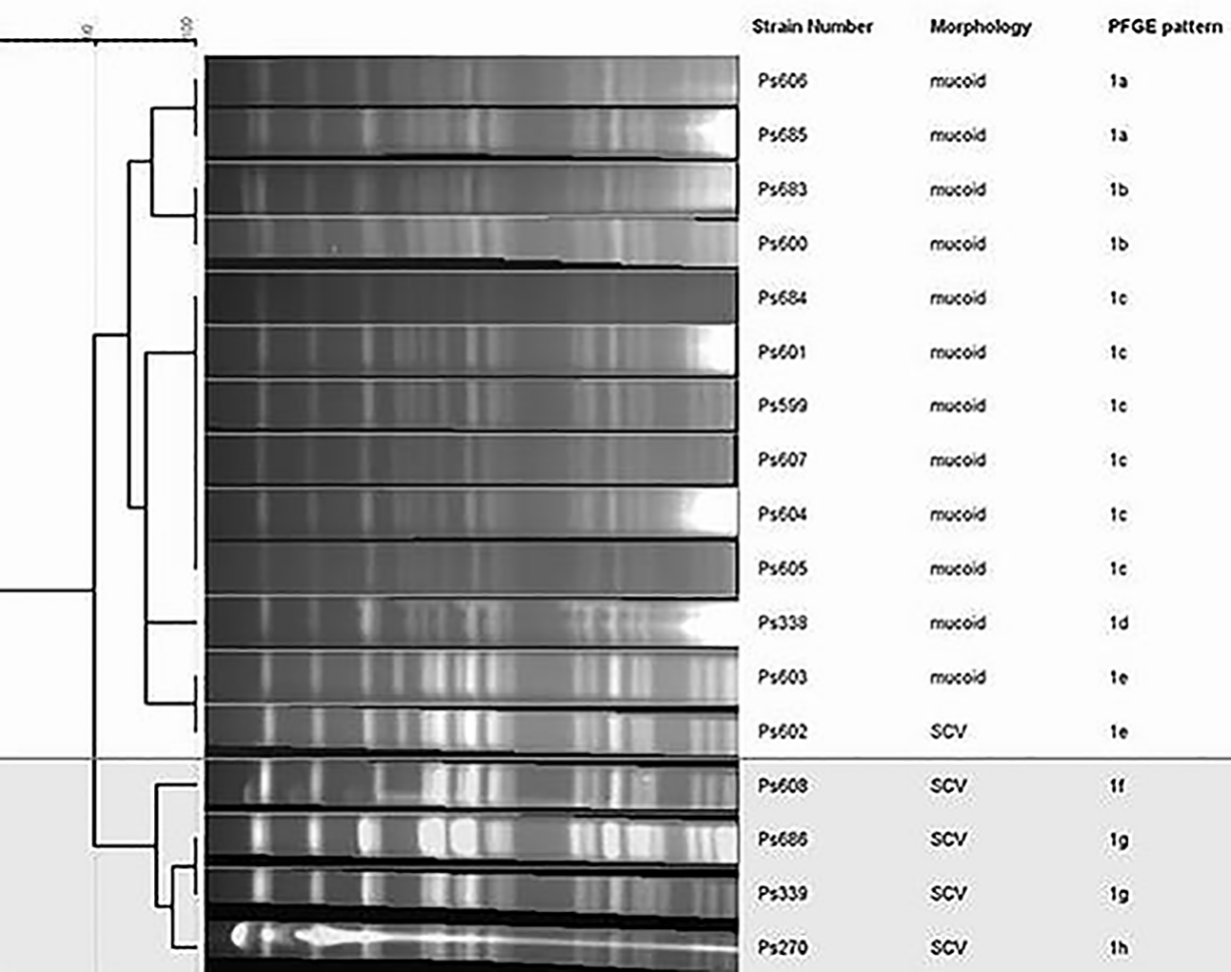
Dendrogram of PFGE patterns in *P*. *aeruginosa* isolates (Java program GelJ). The two main groups are marked.

All *P*. *aeruginosa* isolates belonged to ST412, and had closely related PFGE patterns (only differed by ≤3 bands), classified in eight subtypes (from 1a to 1h) (Table 1, Fig 1). According to the dendogram, two main groups were observed: one includes all mucoid isolates and one SCV isolate (subtypes 1a-1e), and the other group corresponds to four SCV isolates (subtypes 1f-1h). Remarkably, one mucoid isolate (Ps603) showed identical PFGE pattern (1e) to one SCV isolate (Ps602). Regarding serotype identification, all isolates were polyagglutinable (i. e., agglutinated in two or more antisera), except one of them which was autoagglutinable (Ps608).

### Antimicrobial susceptibility

All SCV isolates showed resistance to imipenem, doripenem, netilmicin and diminished susceptibility to ticarcillin, piperacillin, piperacillin/tazobactam, ceftazidime, cefepime, and gentamicin. One mucoid isolate (Ps603) presented decreased susceptibility to imipenem, ticarcillin, piperacillin, piperacillin/tazobactam, ceftazidime, cefepime, and other two mucoid isolates (Ps606 and Ps683) to imipenem. The remaining mucoid isolates were susceptible to all antimicrobials tested (data included in S2 Table). None of *P*. *aeruginosa* isolates exhibited ESBL, MBL, or class A carbapenemase phenotypes. AmpC hyperproduction was detected in all isolates.

### Study of efflux RND pumps

All isolates showed the same alterations in genes related to efflux pumps (Table 2). *mexAB* and *mexEF* genes were identical to those of PAO1 strain, but some of their regulatory genes, as well as *mexCD* and *mexXY*, presented amino acid changes. All our isolates were temocillin susceptible, and PAβN (20 mg/L) increased the susceptibility to ciprofloxacin/norfloxacin in all isolates, and to imipenem/meropenem only among SCVs.

**Table 2:** Molecular characterization of efflux RND pumps and their regulators in SCV and mucoid *P*. *aeruginosa* isolates.

Efflux RND pumps	Efflux pump and regulatory genes (type)	Amino acid changes
MexAB-OprM	*mexA*	none
	*mexB*	none
	*mexR* (repressor)	none
	*nalC* (repressor)	G71E, S209R
	*nalD* (repressor)	none
MexCD-OprJ	*mexC*	F118S
	*mexD*	P809A†
	*nfxB* (repressor)	none
MexEF-OprN	*mexE*	none
	*mexF*	none
	*mexS* (repressor)	D249N
	*mexT* (activator)	Shortened protein (Δ8 nt 235–242)
MexXY-OprM	*mexX*	K329Q, L331V, W358R
	*mexY*	I536V, T543A, V980I
	*mexZ* (repressor)	none

†In Ps603 isolate the amino acid change S845A was also detected.

### Characterization of porin OprD

All SCVs showed the same OprD sequence, while three different sequences were identified among mucoid isolates (Table 3). Three common substitutions (Asp43Asn, Ser57Glu, Ser59Arg) were detected in the 17 isolates. Frameshifts and stop codons were identified among imipenem-resistant isolates. Eleven additional common changes were found among mucoid isolates, and one more (Leu11Gln) was observed in two imipenem-intermediate isolates as compared to the susceptible ones. The promoter regions of all isolates were identical to those of PAO1. No porin OprD band was detected by SDS-PAGE in those isolates harboring shorter OprD proteins (189 and 326 amino acids), and the values of *oprD* mRNA expression were lower or very similar to that of PAO1. In the remaining isolates, a band corresponding to the porin was detected, and *oprD* mRNA expression level was almost double than that measured for PAO1 (Table 3).

**Table 3:** Molecular characterization of porin OprD in SCV and mucoid *P*. *aeruginosa* isolates.

	Isolate	MIC (mg/L) of imipenem	OprD size (aa)	Amino acid changes in OprD sequence	Insertion/deletion	OprD loops affected	OprD expression†	2^-ΔΔCt^ (OprD)‡
**SCV isolates**	Ps270, Ps339, Ps602, Ps608, Ps686	>32	189	D43N, S57E, S59R	Deletion of 11 bp at codon 130 (nt 390)	L1	No	0.45±0.16
**Mucoid isolates**	Ps338, Ps599, Ps600, Ps601, Ps604, Ps605, Ps607, Ps684, Ps685	0.25–1.5	441	D43N, S57E, S59R, E202Q, I210A, E230K, S240T, N262T, A267S, A281G, K296Q, Q301E, R310G, V359L	Loop 7-short	L1, L4, L5, L6, L7	Yes	1.85±0.71
	Ps606, Ps683	4–6	441	L11Q, D43N, S57E, S59R, E202Q, I210A, E230K, S240T, N262T, A267S, A281G, K296Q, Q301E, R310G, V359L	Loop 7-short	L1, L4, L5, L6, L7	Yes	1.99±0.29
	Ps603	6	326	D43N, S57E, S59R, E202Q, I210A, E230K, S240T, N262T, A267S, A281G, K296Q, Q301E, R310G, Q327STOP		L1, L4, L5, L6	No	1.17

†Determined by SDS-PAGE of outer membrane proteins

‡*oprD* mRNA expression determined by RT-qPCR. Relative gene expression was calculated by 2^-ΔΔCT^ method. The *rpsL* gene was used as reference and *P*. *aeruginosa* PAO1 strain as calibrator (value of *P*. *aeruginosa* PAO1 = 1).

### Determination of integron structures

**Fig 2:**
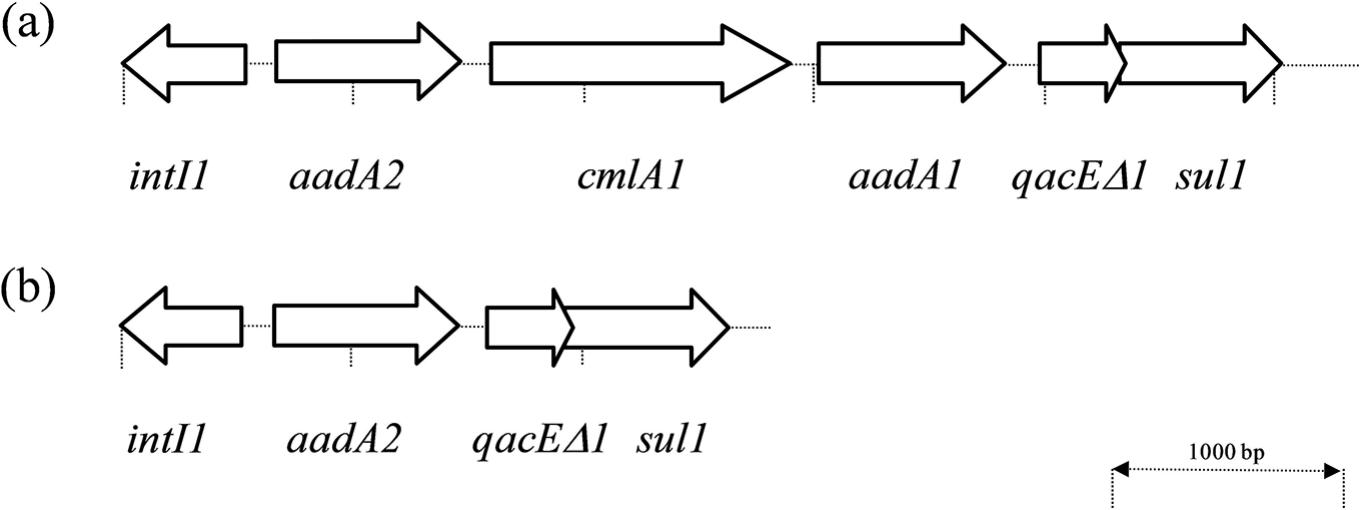
Representation of class 1 integron arrays found among our *P*. *aeruginosa* isolates. The position and orientation of the gene cassettes are indicated by arrows. *intI1*: integrase gene; *aadA*: aminoglycoside 3'-adenyltransferase gene (resistance to streptomycin and spectinomycin); *cmlA*: chloramphenicol acetyltransferase gene (resistance to chloramphenicol); *qacE*: quaternary ammonium compound resistance gene; *sul1*: sulphonamide resistant dihydropteroate synthase gene. (a) new integron structure (In1342) found in four SCVs (Ps270, Ps339, Ps602, Ps686) and three mucoid isolates (Ps603, Ps606, Ps683) [Sequence data of this integron is available in the GenBank database with accession number MF135190 (Ps270 isolate)]. (b) integron In127 structure found in all *P*. *aeruginosa* isolates except in Ps608.

All but one (Ps608) isolates harbored class 1 integrons. Four SCV isolates and three mucoid isolates contained two class 1 integrons (In127 and In1342) (Fig 2). This study is the first to describe the In1342 gene cassette arrangement (GenBank accession number MF135190). The remaining isolates harbored only the In127 class 1 integron (Fig 2). PcW promoter was identified in all integrons.

### Presence of virulence and DNA mismatch repair system genes

All isolates contained the *exoS*, *exoY*, *exoT*, *exoA*, *lasA*, *lasB*, *aprA*, *rhlAB*, *rhlI*, *rhlR*, *lasI*, and *lasR* genes. No *exoU* gene was detected among our isolates. Moreover, no amino acid changes were identified in MutL and MutS proteins.

### Phenotypic assays and gene expression

**Fig 3:**
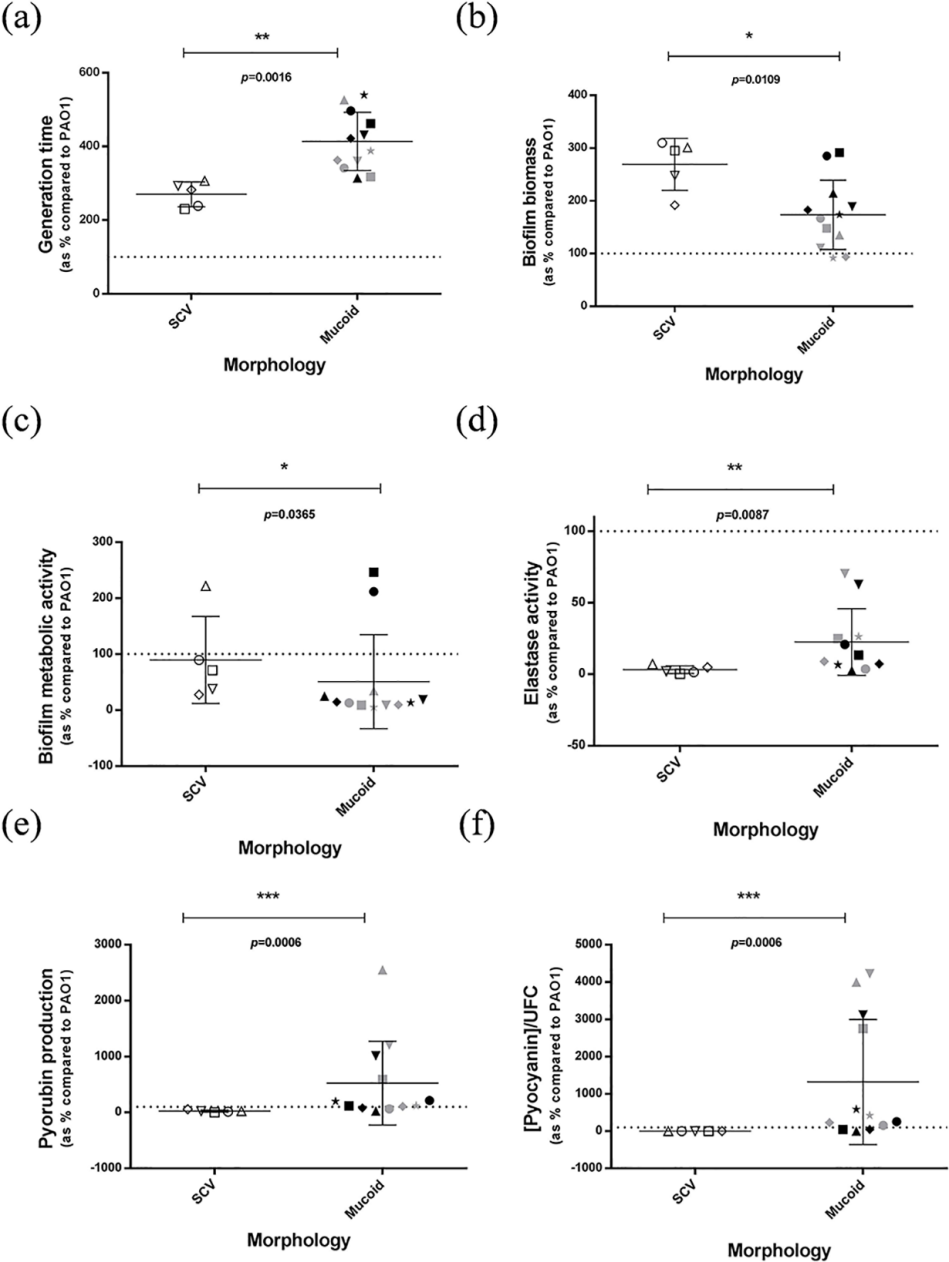
Growth and phenotypic assays of SCV and mucoid isolates. (a) Generation times (time taken for the doubling of population). (b) Biofilm biomass determined by staining with crystal violet. (c) Metabolic activity of bacteria within biofilm determined by staining with fluorescein diacetate. (d) Elastase assay (in this case, Ps600 was eliminated due to its high capacity of pyorubin production). (e) Pyorubin production assay. (f) Pyocyanin production assay. *p≤0.05, **p≤0.01, ***p≤0.001. Dotted line (PAO1 value = 100%). White triangle up, Ps270; white triangle down, Ps339; white square, Ps602; white circle Ps608; white rhombus, Ps686; grey star, Ps338; grey square, Ps599; grey triangle up, Ps600; grey triangle down, Ps601; black triangle up, Ps603; black rhombus, Ps604; grey circle, Ps605; black square, Ps606; black triangle down, Ps607; black circle, Ps683; black star, Ps684; grey rhombus, Ps686.

**Fig 4:**
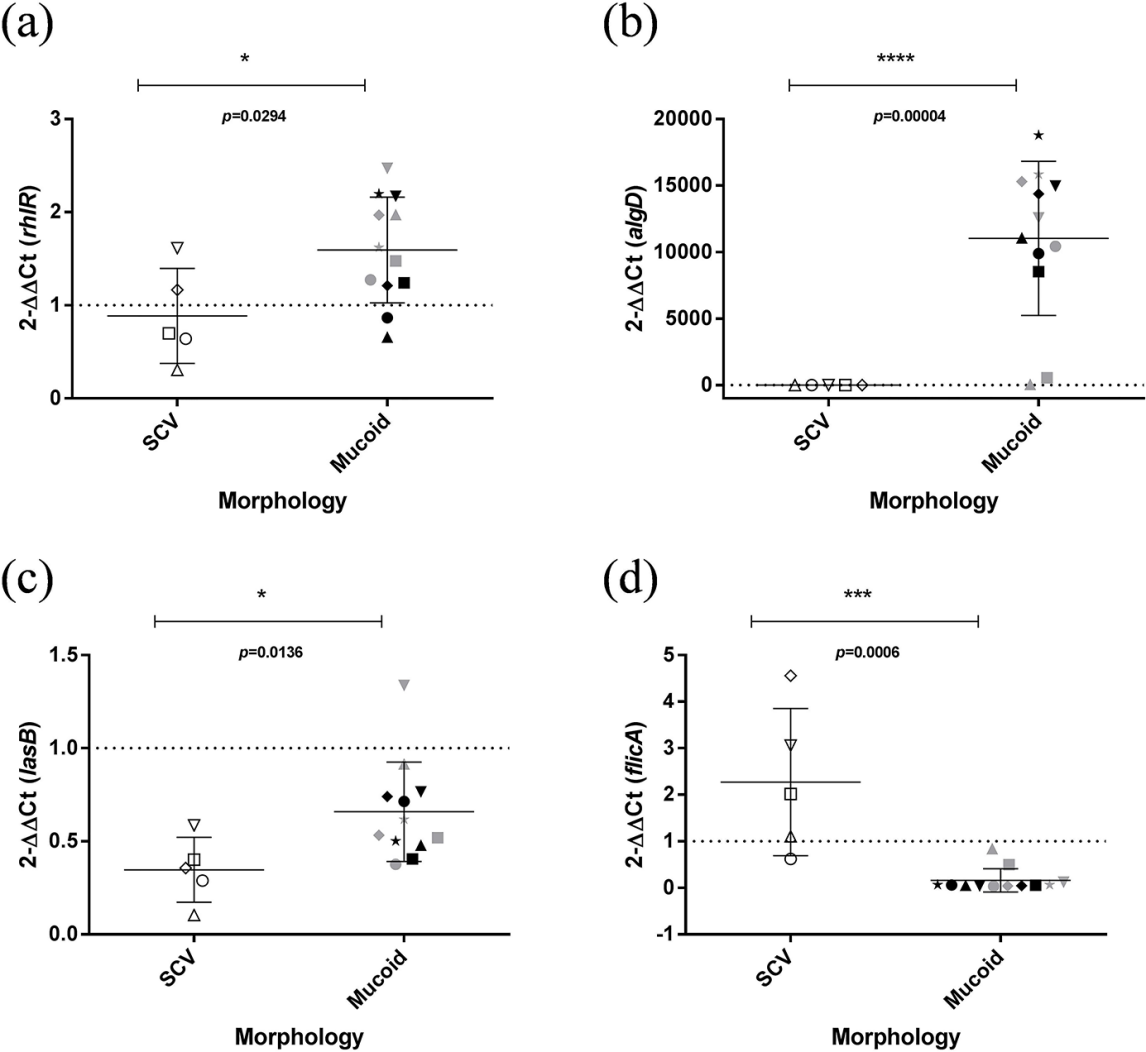
Expression of the genes of SCV and mucoid isolates. (a) *rhlR*, (b) *algD*, (c) *lasB*, and (d) *flicA* expression. *p≤0.05, **p≤0.01, ***p≤0.001, **** *p*≤0.0001. Dotted line (PAO1 value = 1). White triangle up, Ps270; white triangle down, Ps339; white square, Ps602; white circle Ps608; white rhombus, Ps686; grey star, Ps338; grey square, Ps599; grey triangle up, Ps600; grey triangle down, Ps601; black triangle up, Ps603; black rhombus, Ps604; grey circle, Ps605; black square, Ps606; black triangle down, Ps607; black circle, Ps683; black star, Ps684; grey rhombus, Ps686.

The CF isolates grew slower than *P*. *aeruginosa* PAO1, SCV growing significantly faster than mucoid isolates (p = 0.0016) (Fig 3). Statistically significant differences between SCV and mucoid isolates were identified in biofilm quantification, pigment production and elastase assays (Fig 3). All our isolates showed low swimming and swarming motility and no statistically significant differences were identified (S2 Table and S1 Fig). S3 Table shows RT-qPCR results of SCV and mucoid isolates. All isolates showed reduced expression of *lasB*, *pcrV*, *popB* and *popD* genes in comparison with PAO1. Fig 4 shows the expression of the virulence genes for which statistically significant differences were detected between SCV and mucoid isolates. Moreover, some statistically significant correlations among these phenotypic assay results and/or gene expressions were detected (S4 Table).

## Discussion

*P*. *aeruginosa* isolates successively collected from the same CF patient over a 3 year period were characterized. All isolates from this patient showed closely related PFGE patterns and the same ST (ST412). The use of both PFGE and MLST techniques has been recommended for typing *P*. *aeruginosa* involved in chronic processes [30]. Recently, the first Spanish multi-center study on the CF microbiology has been published [31], but no isolate was ascribed either to international epidemic clones or to ST412 clone. Only four ST412 isolates have been registered in MLST database (one from a shower, two from humans and one from unknown origin) (http://pubmlst.org/paeruginosa/). Additionally, ST412 was previously described in one nosocomial carbapenem-resistant isolate (P179) isolated from a blood sample in Korea [32]. The *exoS*, *exoY*, *exoT*, and *exoA* genes and less biofilm production with respect to PAO1 strain were detected in P179 isolate as well as in our CF isolates, which also amplified *lasA* and *aprA* genes that were absent in P179 [32].

CF *P*. *aeruginosa* infections typically progress from the acquisition of a single environmental strain to an extensive genetic and phenotypic adaptation to the lung environment [6]. Chronic infections are commonly caused by a single *P*. *aeruginosa* lineage. However, different lineages have been found in isolates from the same sputum sample or obtained longitudinally from the same patient [33–35]. Due to the high intraspecific *P*. *aeruginosa* diversity in the CF lung, caution is required when assuming that one or few isolates are the cause of an infection in the CF patient. In addition, the prevalence and persistence of the different morphotypes depend on each patient and environmental selection [36].

In our work, SCV and mucoid *P*. *aeruginosa* colony morphologies were identified, that are the most commonly found in CF patients [6,8]. Furthermore, most of them were mucoid (12/17 isolates) agreeing with others [37]. Our SCV and mucoid isolates were slow-growing and high biofilm-producing (biomass), had reduced their expression of toxins and their quorum sensing, and showed a low motility. All of these characteristics are typical of *P*. *aeruginosa* obtained in chronic infections [6].

Even so, important differences were found between both groups. Although both morphotypes have been associated with resistance, it has been hypothesized that SCVs are selected by prolonged antibiotic treatment [6]. Our SCVs were more antimicrobial resistant than mucoid isolates, in accordance with previous studies [38,39]. Hypersusceptibility has been identified in *P*. *aeruginosa* from CF patients in which resistant strains were also found [40].

β-lactam resistance in *P*. *aeruginosa* is usually due to the increased AmpC activity, and the expression of several efflux systems. All our isolates showed AmpC hyperproduction and overexpressed *ampC* gene. However, only the five SCVs and one mucoid isolate (Ps603) presented diminished susceptibility to β-lactams. Discordances between antibiogram profiles and known resistance mechanisms have been associated with CF *P*. *aeruginosa* [40,41], such as *ampC*-overexpressing strains that were susceptible to third generation cephalosporins or monobactams [41]. Indeed it has been observed that strains with a deleted MexAB-OprM pump (affecting β-lactam efflux), are susceptible to these antibiotics even when these strains overexpressed *ampC* [42]. Likewise, susceptibility to temocillin has been associated with mutations in MexAB-OprM in CF *P*. *aeruginosa* [43]. All our isolates were susceptible to temocillin, whereas no mutations were identified in *mexA*/*mexB* genes but two amino acid changes were identified in the regulator NalC. When PAβN was used, increased susceptibility to ciprofloxacin/norfloxacin was identified in all isolates and to imipenem/meropenem only in SCVs. According to these results seems that MexEF-OprN pump was active among our CF isolates.

It has been suggested that nucleotide insertions or deletions in *oprD* gene are the main changes leading to loss of this porin and to imipenem resistance phenotypes [44]. In our case, the isolates that lacked the band corresponding to the porin OprD were resistant (SCVs) or intermediate (Ps603) to imipenem. In the remaining isolates, imipenem susceptibility was observed except in Ps606 and Ps683, in which the change Leu11Gln was found. This carbapenem resistance diversity might have relevant clinical consequences in diagnostic laboratories.

In many cases, routine microbiology testing does not predict response to therapy in CF patients [45], being particularly important in the case of chronic *P*. *aeruginosa* infections. The different subpopulations and antimicrobial susceptibility patterns detected among our *P*. *aeruginosa* CF isolates might be a critical factor in the lack of correlation between microbiology testing data and clinical outcomes. In diagnostic laboratories only one or two colonies are usually tested, and many automatic testing methods are used. The coexistence of several subpopulations of this microorganism could be undervalued. Moreover, the slow growth of these isolates can give mistaken results when automatic susceptibility testing methods are used. The use of disc-diffusion agar methods, in addition to these automatic techniques, seems advisable in these cases to detect the different morphotypes [46]. Agar diffusion methods have demonstrated to be effective to test CF isolates although it seems that are more reliable for non-mucoid than for mucoid isolates [47]. Moreover, conventional clinical microbiological testing only involves the culture of planktonically growing bacteria, without considering the resistance due to biofilm formations [4]. Several possible changes have been proposed, such as revise susceptibility breakpoints for CF isolates, use a more CF relevant media or use molecular methods for detection of resistance mechanisms [46].

Our mucoid isolates showed an elevated expression of *algD*, which was highly expected. Interestingly, most of mucoid isolates produced high amounts of pyorubin and pyocyanin and showed elevated elastase activity. This unusual phenotype, in which an overexpression of genes encoding pigments and elastase is detected, has been observed in CF patients, and has been related to exacerbation periods [48]. Remarkably, Ps607 that was obtained when the patient was with hypercapnic coma, showed a very high elastase activity and elevated pyorubin and pyocyanin production.

SCVs are known as slow-growing isolates, excellent biofilm formers, and important exopolysaccharide producers [49]. Our SCV isolates produced high levels of biofilm compared with those of PAO1 and mucoid ones. SCV isolates grew slower than PAO1, but faster than mucoid isolates as was previously observed [50]. Regarding exopolysacharides production, the overexpression of the genes *pel* and *psl* has been linked to SCV formation, leading to hyperadherence and hyperaggregation [8]. This was not confirmed here, as the expression of *pelA* and *pslA* genes was similar in both SCV and mucoid isolates.

No differences among SCV and mucoid isolates were also identified in toxin gene expression, being very low in both morphotypes. The same occurred with motility, although the expression of *flicA* was higher in SCVs than mucoid isolates. A correlation between flagellin expression level and motility was not detected by us and by others [24]. Moreover, despite the high occurrence of hypermutator CF lineages and its role in the evolution of *P*. *aeruginosa* during chronic respiratory infections [5], our isolates did not present amino acid changes in MutL and MutS.

Analyzing the data in depth, neither all SCVs nor all mucoid isolates were equal among them. Interestingly, the Ps608 SCV did not contain any integron and this isolate was the only one obtained from one tracheal aspirate sample. Furthermore, some mucoid isolates (Ps603, Ps606, Ps683) presented some characteristics more similar to those detected in SCVs. Biofilm biomass of these three isolates was higher than for other mucoid isolates and Ps603 showed high GT, low elastase activity and did not produce much pyorubin and pyocyanin. Just like SCVs, these isolates contained two integrons and were not susceptible to all tested antibiotics. Therefore, the division between different morphotypes may not always be clear.

This study presented as main limitation that the initial isolates of this patient could not be included. A comparison between earlier isolates and those studied here would have undoubtedly provided data of great interest on the evolution of this microorganism. However, our objective was focused on the biological factors of these isolates once they have been established chronically, going deeper into the antimicrobial resistance mechanisms that cause unsatisfactory therapeutic outcomes in these patients. The selection of the isolates was therefore adequate, although a greater number, especially of SCV, would have provided a better statistical accuracy. Another important limitation of our results is the applicability to the pediatric CF population due to the age of the patient and the chronicity of infection. This population would be a remarkable target for future research.

The current papers focused on the study of *P*. *aeruginosa* during CF infections usually compare chronic isolates with those detected in early stages or they only analyzed some particular characteristics [7,34,35,48]. Moreover, although some data about antimicrobial phenotypes of these isolates is present in some of them, information about the different antimicrobial resistance mechanisms is scarce. In the present work, data about very diverse phenotypic features including antibiotic resistance, pigments, motility or biofilm formation as well as different resistance mechanisms or the expression of relevant genes were obtained. Important differences between SCV and mucoid isolates were identified. These results are of high interest for better understanding the behavior of chronic *P*. *aeruginosa* isolates in CF patients. Future studies should clarify which are the molecular bases that explain the obtained data for a better understanding of the evolution of these morphotypes.

## Supporting information

S1 Table Sequences of the primers used for RT-qPCR, and for PCR and sequencing of efflux pump genes. (DOC)

S2 Table Phenotypic characteristics of the 17 *P*. *aeruginosa* isolates. (DOCX)

S3 Table mRNA expression (2^-ΔΔCt^) detected in SCV and mucoid isolates. (DOCX)

S4 Table Correlation between the different phenotypic assay results and/or gene expressions for all isolates. (DOCX)

S1 Fig Motility results of *P*. *aeruginosa* PAO1 strain, Ps602 isolate (SCV) and Ps601 isolate (mucoid). (a) Swimming motility. (b) Swarming motility.
(DOCX)
