# Supplementary material for: Great phenotypic and genetic variation among successive chronic Pseudomonas aeruginosa from a cystic fibrosis patient
Source: PLoS One. 2018 Sep 13;13(9):e0204167. doi: 10.1371/journal.pone.0204167 (PMC6136817; doi:10.1371/journal.pone.0204167)
Supplement: S1 Table — (DOC) [file pone.0204167.s001.doc]

S1 Table. Sequences of the primers used for RT-qPCR and PCR and sequencing of efflux pump genes

| **Gene** | **Primer name** | **Primer sequence (5'-3')** |
| --- | --- | --- |
| *rpsL* | qrpsL-F | CGGCACTGCGTAAGGTATGC |
|  | qrpsL-R | CGTACTTCGAACGACCCTGCT |
| *ampC* | qampC-F | CTGTTCGAGATCGGCTC |
|  | qampC-R | CGGTATAGGTCGCGAG |
| *oprD* | qoprD-F | TCCGCAGGTAGCACTCAGTTC |
|  | qoprD-R | AAGCCGGATTCATAGGTGGTG |
| *algD* | qalgD-F | GCGACCTGGACCTGGGCT |
|  | qalgD-R | TCCTCGATCAGCGGGATC |
| *rhlR* | qrhlR-F | CTGGGCTTCGATTACTACGC |
|  | qrhlR-R | CCCGTAGTTCTGCATCTGGT |
| *lasR* | qlasR-F | ACGCTCAAGTGGAAAATTGG |
|  | qlasR-R | TCGTAGTCCTGGCTGTCCTT |
| *lasB* | qlasB-F | AAGCCATCACCGAAGTCAAG |
|  | qlasB-R | CGGATCACCAGTTCCACTTT |
| *pslA* | qpslA-F | AAGATCAAGAAACGCGTGGAAT |
|  | qpslA-R | TGTAGAGGTCGAACCACACCG |
| *pelA* | qpelA-F | CCTTCAGCCATCCGTTCTTCT |
|  | qpelA-R | TCGCGTACGAAGTCGACCTT |
| *exoS* | qexoS-F | GGCGGATGCGGQAAAAGTAC |
|  | qexoS-R | CTGACGCAGAGCGCGATT |
| *exoT* | qexoT-F | ATGCGGTAATGGACAAGGTC |
|  | qexoT-R | CTGGTACTCGCCGTTGGTAT |
| *pcrV* | qpcrV-F | CGATGAGTACCCCTTCGAGA |
|  | qpcrV-R | ATTTCTGGATGAAGCGGTTG |
| *popB* | qpopB-F | CTTTGGTTGGATCAGTGCAA |
|  | qpopB-R | CCGAGCTTTTCCATCACTTC |
| *popD* | qpopD-F | ACACGGTGATTCCAGTCCTTC |
|  | qpopD-R | CTGGTTATGGCTCTGGGTGT |
| *flicA* | qflicA-F | GAACGTGGCTACCAAGAACG |
|  | qflicA-R | GTGGTGGTGTTGCTGATACG |
| *mexA* | mexA -F | GCGAGGCTTTCGGACGTTTA |
|  | mexA-R | GGCAGACTGAGGATCGACA |
| *mexB* | mexB-F | CAAGGGGATTCGTAATGTC |
|  | mexB-R | GTGAACATCCAGATCATCC |
| *mexC* | mexC-F | ATTTGCGTGCAATAGGAAGG |
|  | mexC-R | CTTCGGCACGATCTTCACC |
| *mexD* | mexD-F | GTAGCGCAGTACCCCAATGT |
|  | mexD-R | AGCAGCCAGACGAAACAGAT |
| *mexE* | mexE-F | AACAACCGCTGAACGAGTG |
|  | mexE-R | GCGGTAGACGGTCTTGTTGT |
| *mexF* | mexF-F | CTGACCCTGACCATCACCTT |
|  | mexF-R | GAAGCCGTTGTAGTGCATCA |
| *mexX* | mexX-F | CCTGGTCGCCCTATTCCT |
|  | mexX-R | GTTCTCGACGATCACCCACT |
| *mexY* | mexY-F | CAGCATCCAGCTGATCGTTA |
|  | mexY-R | AACAGCGGTACCAGGAACAC |
| *mexR* | mexR-F | CCAGTAAGCGGATAC |
|  | mexR-R | GGATGATGCCGTTCACCTG |
| *nalC* | nalC-F | TCAACCCTAACGAGAAACGCT |
|  | nalC-R | TCCACCTCACCGAACTGC |
| *nalD* | nalD-F | GCGGCTAAAATCGGTACACT |
|  | nalD-R | ACGTCCAGGTGGATCTTGG |
| *nfxB* | nfxB-F | CGCCCCGATCCTTCCTATT |
|  | nfxB-R | ACGAGCGTCACGGTCCTTT |
| *mexS* | mexS-F | ATACAGTCACAACCCATGA |
|  | mexS-R | TCAACGATCTGTGGATCT |
| *mexT* | mexT-F | TGCATCACGGGGTGAATAAC |
|  | mexT-R | GGTAGCGCCAGGAGAAGTG |
| *mexZ* | mexZ-F | CCAGCAGGAATAGGGCGACCAGGGC |
|  | mexZ-R | CAGCGTGGAGATCGAAGGCAGCCGG |
