# Supplementary material for: Great phenotypic and genetic variation among successive chronic Pseudomonas aeruginosa from a cystic fibrosis patient
Source: PLoS One. 2018 Sep 13;13(9):e0204167. doi: 10.1371/journal.pone.0204167 (PMC6136817; doi:10.1371/journal.pone.0204167)
Supplement: S2 Table — (DOCX) [file pone.0204167.s002.docx]

**S2 Table. Phenotypic characteristics of the 17 *P. aeruginosa* isolates**.

|  | **Isolate** | **Resistance phenotype**† | **Generation Time (min)** | **AmpC hyperproduction (CLOX 250-CAZ)**† | **Efflux pump overexpression (PAβN 20)**† | | | **Biofilm**  **% of PAO1** | | **Elastase % of PAO1** | **Pyorubin % of PAO1** | **[Pyocyanin] % of PAO1** | **Motility % of PAO1** | |
| --- | --- | --- | --- | --- | --- | --- | --- | --- | --- | --- | --- | --- | --- | --- |
|  |  |  |  |  | **CIP** | **IPM** | **MEM** | **CV** | **FDA** |  |  |  | **swimming** | **swarming** |
| **SCV isolates** | Ps270 | IPM, DOR, NET, TIC^i^, PIP^i^, TZP^i^, CAZ^i^, FEP^i^, GEN^i^ | 59.24 | Yes | Yes | Yes | No | 301.03 | 221.74 | 7.20 | 25.22 | 1.35 | 12.10 | 7.04 |
|  | Ps339 | IPM, DOR, NET, TIC^i^, PIP^i^, TZP^i^, CAZ^i^, FEP^i^, GEN^i^ | 56.59 | Yes | Yes | Yes | No | 247.99 | 37.91 | 2.13 | 23.61 | 0.95 | 13.77 | 7.43 |
|  | Ps602 | IPM, DOR, NET, TIC^i^, PIP^i^, TZP^i^, CAZ^i^, FEP^i^, GEN^i^ | 44.51 | Yes | Yes | Yes | No | 295.14 | 71.31 | 0.36 | 3.29 | 0.26 | 20.10 | 6.02 |
|  | Ps608 | IPM, DOR, NET, TIC^i^, PIP^i^, TZP^i^, CAZ^i^, FEP^i^, GEN^i^ | 46.15 | Yes | Yes | Yes | No | 309.92 | 89.58 | 1.52 | 14.04 | 0.44 | 22.01 | 5.36 |
|  | Ps686 | IPM, DOR, NET, TIC^i^, PIP^i^, TZP^i^, CAZ^i^, FEP^i^, GEN^i^ | 54.55 | Yes | Yes | Yes | No | 191.93 | 27.66 | 4.83 | 57.65 | 2.70 | 26.20 | 7.13 |
| **Mucoid isolates** | Ps338 | Susceptible | 75.03 | Yes | Yes | No | No | 91.80 | 4.68 | 26.35 | 120.10 | 423.69 | 15.17 | 10.80 |
|  | Ps599 | Susceptible | 61.37 | Yes | Yes | No | No | 147.92 | 8.87 | 25.01 | 590.40 | 2753.00 | 16.45 | 7.07 |
|  | Ps600 | Susceptible | 101.71 | Yes | Yes | No | No | 135.09 | 34.44 | 640.52 | 2551.54 | 3997.05 | 18.52 | 7.15 |
|  | Ps601 | Susceptible | 69.59 | Yes | Yes | No | No | 110.78 | 8.86 | 70.44 | 1208.25 | 4227.74 | 38.20 | 22.59 |
|  | Ps603 | IPM^i^, TIC^i^, PIP^i^, TZP^i^, CAZ^i^, FEP^i^ | 60.74 | Yes | Yes | No | No | 214.34 | 24.94 | 2.59 | 25.27 | 2.03 | 16.84 | 5.24 |
|  | Ps604 | Susceptible | 81.53 | Yes | Yes | No | No | 182.99 | 14.63 | 7.31 | 81.27 | 45.56 | 15.10 | 6.32 |
|  | Ps605 | Susceptible | 65.92 | Yes | Yes | No | No | 166.73 | 12.83 | 3.67 | 65.26 | 159.07 | 16.57 | 7.57 |
|  | Ps606 | IPM^i^ | 89.37 | Yes | Yes | No | No | 291.57 | 246.50 | 13.35 | 119.36 | 45.18 | 12.72 | 5.36 |
|  | Ps607 | Susceptible | 83.36 | Yes | Yes | No | No | 189.02 | 18.74 | 62.87 | 1016.58 | 3122.61 | 14.24 | 6.54 |
|  | Ps683 | IPM^i^ | 96.00 | Yes | Yes | No | No | 285.12 | 211.78 | 20.87 | 214.40 | 255.56 | 13.77 | 7.40 |
|  | Ps684 | Susceptible | 104.32 | Yes | Yes | No | No | 173.99 | 13.04 | 6.65 | 198.41 | 586.15 | 13.97 | 6.72 |
|  | Ps685 | Susceptible | 70.10 | Yes | Yes | No | No | 93.89 | 9.49 | 9.02 | 109.86 | 233.38 | 15.25 | 6.27 |

† IPM: imipenem; DOR: doripenem; NET: netilmicin; TIC: ticarcillin; PIP: piperacillin; TZP: piperacillin-tazobactam; CAZ: ceftazidime; FEP: cefepime; GEN: gentamicin; CLOX: cloxacillin; CIP: ciprofloxacin; MEM: meropenem ^i^: intermediate
