# Supplementary material for: Great phenotypic and genetic variation among successive chronic Pseudomonas aeruginosa from a cystic fibrosis patient
Source: PLoS One. 2018 Sep 13;13(9):e0204167. doi: 10.1371/journal.pone.0204167 (PMC6136817; doi:10.1371/journal.pone.0204167)
Supplement: S3 Table — (DOCX) [file pone.0204167.s003.docx]

**S3 Table. mRNA expression (2^-ΔΔCt^) detected in SCV and mucoid isolates.**

| **Gene** | **SCV isolates**† | **Mucoid isolates**† | ***p* value (SCV *vs* mucoids)** |
| --- | --- | --- | --- |
| ***ampC*** | 702.37±394.31 | 1170.88±1220.42 | 0.7214 |
| ***oprD*** | 0.45±0.16 | 1.82±0.65 | <0.0001 |
| ***algD*** | 3.29±1.02 | 11029.68±5790.27 | <0.0001 |
| ***rhlR*** | 0.89±0.51 | 1.59±0.57 | 0.0294 |
| ***lasR*** | 5.91±2.87 | 7.21±3.96 | 0.7214 |
| ***lasB*** | 0.35±0.17 | 0.66±0.27 | 0.0136 |
| ***pslA*** | 2.35±1.73 | 2.34±0.84 | 0.9874 |
| ***pelA*** | 1.93±0.77 | 1.87±1.69 | 0.6445 |
| ***exoS*** | 1.66±0.53 | 1.97±0.55 | 0.3077 |
| ***exoT*** | 4.55±1.64 | 6.53±2.40 | 0.1147 |
| ***pcrV*** | 0.54±0.13 | 0.61±0.15 | 0.2786 |
| ***popB*** | 0.34±0.13 | 0.39±0.11 | 0.3846 |
| ***popD*** | 0.81±0.47 | 0.98±0.26 | 0.3410 |
| ***flicA*** | 2.27±1.58 | 0.16±0.25 | <0.0001 |

† Relative gene expression was calculated by 2^-ΔΔCT^ method. The *rpsL* gene was used as reference and *P. aeruginosa* PAO1 strain as calibrator, except in the case of *flicA* in which *P. aeruginosa* CHA strain was used as calibrator (value of *P. aeruginosa* PAO1 or *P. aeruginosa* CHA=1)
