# Supplementary material for: Great phenotypic and genetic variation among successive chronic Pseudomonas aeruginosa from a cystic fibrosis patient
Source: PLoS One. 2018 Sep 13;13(9):e0204167. doi: 10.1371/journal.pone.0204167 (PMC6136817; doi:10.1371/journal.pone.0204167)
Supplement: S4 Table — (DOCX) [file pone.0204167.s004.docx]

**S4 Table. Correlation between the different phenotypic assay results and/or gene expressions for all isolates.**

|  | **all isolates**† |
| --- | --- |
| *lasB* expression vs elastase activity | rho=0.68 |
|  | ***p*=0.0051** ** |
| *rhlR* expression vs [pyocyanin] | rho=0.80 |
|  | ***p*=0.0001** *** |
| *rhlR* expression vs Pyorubin production | rho=0.73 |
|  | ***p*=0.0012** ** |
| *lasR* expression vs elastase activity | rho=0.21 |
|  | *p*=0.4297 |
| *lasR* expression vs *lasB* expression | rho=0.48 |
|  | *p*=0.0557 |
| *lasR* expression vs [pyocyanin] | rho=0.13 |
|  | *p*=0.6254 |
| *lasR* expression vs Pyorubin production | rho=0.08 |
|  | *p*=0.7584 |
| Pyorubin vs pyocyanin production | rho=0.97 |
|  | *p*<0.0001**** |
| Biofilm biomass vs *lasR* expression | rho=-0.23 |
|  | *p*=0.3774 |
| Biofilm biomass vs *algD* expression | rho=-0.65 |
|  | ***p*=0.0054** ** |
| Biofilm biomass vs *rhlR* expression | r=-0.74 |
|  | ***p*=0.0006** *** |
| Biofilm biomass vs *flicA expression* | rho=0.32 |
|  | *p*=0.2159 |
| Biofilm biomass vs *pslA* expression | r=-0.20 |
|  | *p*=0.4506 |
| Biofilm biomass vs *pelA* expression | rho=0.22 |
|  | *p*=0.4042 |
| Biofilm metabolic activity vs *lasR* expression | rho=-0.38 |
|  | *p*=0.1387 |
| Biofilm metabolic activity vs *algD* expression | rho=-0.67 |
|  | ***p*=0.0044** ** |
| Biofilm metabolic activity vs *rhlR* expression | rho=-0.63 |
|  | ***p*=0.0075** ** |
| Biofilm metabolic activity *vs flicA* expression | rho=0.32 |
|  | *p*=0.2122 |
| Biofilm metabolic activity vs *pslA* expression | rho=-0.21 |
|  | *p*=0.4264 |
| Biofilm metabolic activity vs *pelA* expression | rho=0.12 |
|  | *p*=0.6458 |
| Biofilm biomass vs biofilm metabolic activity | rho=0.89 |
|  | *p*<0.0001**** |
| *exoS* expression vs *exoT* expression | rho=0.77 |
|  | ***p*=0.0004** *** |
| *exoS* expression vs *pcrV* expression | rho=0.10 |
|  | *p*=0.7013 |
| *exoT* expression vs *pcrV* expression | r=0.24 |
|  | *p*=0.3597 |
| *popB* expression vs *popD* expression | r=0.62 |
|  | ***p*=0.0081** ** |
| *exoS* expression vs *popB* expression | rho=0.10 |
|  | *p*=0.7084 |
| *exoS* expression vs *popD* expression | rho=0.32 |
|  | *p*=0.2122 |
| *exoT* expression vs *popB* expression | r=0.32 |
|  | *p*=0.2130 |
| *exoT* expression vs *popD* expression | r=0.32 |
|  | *p*=0.2033 |
| *pcrV* expression vs *popB* expression | r=0.35 |
|  | *p*=0.1625 |
| *pcrV* expression vs *popD* expression | r=0.15 |
|  | *p*=0.5737 |
| *exoS* expression vs *rhlR* expression | r=0.53 |
|  | ***p*=0.0301** * |
| *exoS* expression vs *lasR* expression | rho=0.38 |
|  | *p*=0.1281 |
| *exoT* expression vs *rhlR* expression | r=0.43 |
|  | *p*=0.0813 |
| *exoT* expression vs *lasR* expression | rho=0.15 |
|  | *p*=0.5659 |
| *pcrV* expression vs *rhlR* expression | r=-0.21 |
|  | *p*=0.4248 |
| *pcrV* expression vs *lasR* expression | rho=0.22 |
|  | *p*=0.3880 |
| *popB* expression vs *rhlR* expression | r=0.17 |
|  | *p*=0.5098 |
| *popB* expression vs *lasR* expression | rho=0.26 |
|  | *p*=0.3174 |
| *popB* expression vs *rhlR* expression | r=0.35 |
|  | *p*=0.1655 |
| *popB* expression vs *lasR* expression | rho=0.11 |
|  | *p*=0.6664 |
| *pslA* expression vs *pelA* expression | rho=0.41 |
|  | *p*=0.1063 |
| *flicA* expression vs swimming | rho=0.25 |
|  | *p*=0.3403 |
| *flicA* expression vs swarming | rho=0.17 |
|  | *p*=0.5214 |

†Pearson's correlation (r) or Spearman's rank correlation (rho) coefficients (as appropriate) and *p* value is indicated.
