# Supplementary material for: Great phenotypic and genetic variation among successive chronic Pseudomonas aeruginosa from a cystic fibrosis patient
Source: PLoS One. 2018 Sep 13;13(9):e0204167. doi: 10.1371/journal.pone.0204167 (PMC6136817; doi:10.1371/journal.pone.0204167)
Supplement: S1 Fig — (a) Swimming motility. (b) Swarming motility. (DOCX) [file pone.0204167.s005.docx]

**S1 Fig. Motility results of *P. aeruginosa* PAO1 strain, Ps602 isolate (SCV) and Ps601 isolate (mucoid)**

(a) Swimming motility. (b) Swarming motility**.**
